# Liposomal Formulation of Turmerone-Rich Hexane Fractions from *Curcuma longa* Enhances Their Antileishmanial Activity

**DOI:** 10.1155/2014/694934

**Published:** 2014-06-18

**Authors:** Ana Claudia F. Amaral, Luciana A. Gomes, Jefferson Rocha de A. Silva, José Luiz P. Ferreira, Aline de S. Ramos, Maria do Socorro S. Rosa, Alane B. Vermelho, Igor A. Rodrigues

**Affiliations:** ^1^Laboratório de Plantas Medicinais e Derivados, Departamento de Produtos Naturais, Farmanguinhos, FIOCRUZ, Rua Sizenando Nabuco 100, 21041-250 Manguinhos, RJ, Brazil; ^2^Departamento de Microbiologia Geral, Instituto de Microbiologia Paulo de Góes, UFRJ, 21941-590 Rio de Janeiro, RJ, Brazil; ^3^Laboratório de Cromatografia, Departamento de Química, Universidade Federal do Amazonas (UFAM), Setor Sul, Avenida Rodrigo Otávio 3000, 69077-000 Japiim, Manaus, AM, Brazil; ^4^Departamento de Produtos Naturais e Alimentos, Faculdade de Farmácia, UFRJ, 21941-590 Rio de Janeiro, RJ, Brazil

## Abstract

Promastigote forms of *Leishmania amazonensis* were treated with different concentrations of two fractions of *Curcuma longa* cortex rich in turmerones and their respective liposomal formulations in order to evaluate growth inhibition and the minimal inhibitory concentration (MIC). In addition, cellular alterations of treated promastigotes were investigated under transmission and scanning electron microscopies. LipoRHIC and LipoRHIWC presented lower MIC, 5.5 and 12.5 *μ*g/mL, when compared to nonencapsulated fractions (125 and 250 *μ*g/mL), respectively, and to ar-turmerone (50 *μ*g/mL). Parasite growth inhibition was demonstrated to be dose-dependent. Important morphological changes as rounded body and presence of several roles on plasmatic membrane could be seen on *L. amazonensis* promastigotes after treatment with subinhibitory concentration (2.75 *μ*g/mL) of the most active LipoRHIC. In that sense, the hexane fraction from the turmeric cortex of *Curcuma longa* incorporated in liposomal formulation (LipoRHIC) could represent good strategy for the development of new antileishmanial agent.

## 1. Introduction

Leishmaniasis is a parasitic disease and about 12 million people are infected worldwide with increasing numbers of new cases each year [1]. Also in Brazil new cases of leishmaniasis are reported annually [2]. The chemotherapeutic agents used for the treatment of leishmaniasis such as sodium stibogluconate,* N*-methylglucamine antimoniate, pentamidine, and amphotericin B are not orally active and require a long-term parenteral administration. These agents also present severe side effects such as cardio and renal toxicities and they are expensive [3]. Additionally, parasites of the genus* Leishmania *are increasingly resistant to the available antileishmanial agents; thus there is an urgency to find and identify new compounds that could be active against these parasites. Some advances have taken place in this field, for example, miltefosine, an alkylphospholipid, was registered in India for the treatment of visceral leishmaniasis (kala-azar) in 2002 [4]. Also there has been an increase in the use and diversity of drug delivery systems for the treatment of various diseases, such as cancer [5–8] and parasitic diseases [9–11]. Delivery systems like liposomes, nanoparticles, emulsions, and others have provided important advantages in terms of increasing the solubility of hydrophobic compounds and bioavailability, among other benefits [12].

*Curcuma longa *L. (*C. domestica* Valeton;* Amomum curcuma *Jacq.;* Stissera curcuma *Raeusch.), popularly known as “turmeric” (Zingiberaceae), is a herbaceous plant with laterally branched rhizomes of Asian origin that has a growing reputation for its miraculous powers in the treatment and prevention of various diseases [13]. Several pharmacological properties have been described to this rhizome and pure substances derived directly from it, including anti-inflammatory, antibacterial, antioxidant, and antiparasitic activities [14, 15]. In the treatment of human parasitic diseases, turmeric is one of the most widely used species. Its action spectrum includes uses against schistosomiasis, helminthiasis, bebesiosis, scabies, coccidiosis, giardiasis, malaria, trypanosomiasis, and leishmaniasis as recently reviewed [16]. Some researchers have described the activity of curcuminoids isolated from* C. longa *against* Leishmania major* with IC_50_ values from 22 to 60 *μ*M [16–18]. Thus, the aim of this work was to study the* C. longa* nonpolar fractions, turmeric cortex, and turmeric without cortex, separately, and to investigate their efficacy as hexane fractions and liposomal forms against* Leishmania amazonensis* strains.

## 2. Materials and Methods

### 2.1. Leishmania Culture

The MHOM/BR/76/Ma-5 Raimundo strain of* Leishmania (L.) amazonensis* was axenically maintained by weekly subculturing (passage each 5 or 6 days) in PBHIL medium supplemented with 10% fetal bovine serum (FBS), at 26°C as previously described [19]. The infectiveness of the promastigotes was assured by periodical infection of mice peritoneal macrophages.

### 2.2. Evaluation of Minimum Inhibitory Concentration (MIC)

This assay was carried out in a 96-well microtiter plate where the extracts and their liposomal preparations were serially diluted in duplicates to final test concentrations (1–500 *μ*g/mL). Then 5.0 × 10^5^ promastigote forms of* L. amazonensis* were harvested at the stationary phase and added to each well and the plate was incubated at 26°C for 120 h. After the incubation period, resazurin solution (5 mg/100 mL of phosphate buffer saline, PBS, pH 7.2) was prepared and 25 *μ*L added to each well and incubation continued for a further 2–4 h as described [20]. MIC was considered the lowest concentration of the extracts and their liposomal preparations that completely prevented the growth of* Leishmania in vitro*. Alternatively, parasites treated for 120 h were centrifuged, washed in PBS, and then reincubated in fresh culture medium in order to evaluate the leishmanicidal effect. The lowest concentration able to inhibit parasites growth was considered the MLC. The IC_50_ was determined by logarithmic regression analysis of the data obtained as described above.

### 2.3. Leishmanicidal Activity of Liposomal Preparations

Promastigotes of* L. amazonensis* (10^6^ parasites/mL) were incubated in PBHIL medium in the presence of various concentrations of liposomal preparations, and parasite survival and cell morphology were evaluated daily by optical microscopy. Parasite viability was assessed before and after incubation by motility and by trypan blue exclusion, using a hemocytometer chamber. Growth was determined by counting the cells after 24 and 48 hours of treatment.

### 2.4. Scanning Electron Microscopy (SEM)

Parasites were harvested at the early stationary phase of growth, washed twice with PBS, and treated with MIC and sub-MIC of the liposomal preparations for 24 hours. Cells were washed twice in cacodylate buffer pH 7.0 and then fixed in a solution containing 2.5% glutaraldehyde, 4% paraformaldehyde, and CaCl_2_ (25 mg/mL), placed on the poly-L-lysine-coated coverslip and dehydrated in growing concentrations of ethanol. Parasites were then critical point dried in CO_2_, sputter-coated with gold, and observed in a JEOL 6490LV scanning electron microscope.

### 2.5. Transmission Electron Microscopy (TEM)

Parasites were obtained as described above. After treatment with MIC and sub-MIC for 24 hours, cells were fixed in 2.5% glutaraldehyde in 0.1 M sodium cacodylate buffer containing 3.5% sucrose (pH 7.4 at 4°C for 60 min), postfixed with a 1% osmium tetroxide and potassium ferrocyanide solution for 1 hour, dehydrated sequentially in acetone, and then embedded in Epon 812. Thin sections were cut using an LKB ultramicrotome and collected on copper grids. Sections were stained with uranyl acetate and lead citrate and examined in a JEOL JEM1011 transmission electron microscope.

### 2.6. Plant Material and Extraction Method

The plant rhizome was purchased from the local market and authenticated by comparison with a voucher deposited at the Herbarium of INPA (Amazonas state), under number 224169. The turmeric cortex and turmeric without cortex (120 and 180 g, resp.) were separated and exhaustively macerated in methanol. The extracts were filtered and the solvents were evaporated under reduced pressure to yield 7.4% of methanol crude turmeric cortex extract and 8.2% of methanol turmeric without cortex crude extract. The methanol crude extracts from the turmeric cortex and turmeric without cortex of* C*.* longa* were submitted to liquid-liquid partition in hexane, to yield, relative to the vegetal material (p/p), 0.43% of the hexane fraction from the turmeric cortex (hexRHIC) and 1.19% of the hexane fraction from the turmeric without cortex (hexRHIWC).

### 2.7. GC-MS Analyses

*Sample Preparation*. The hexane fractions were analyzed by gas chromatography coupled to mass spectrometry (GC-MS). For sample analyses and identification of the substances a gas chromatograph (Agilent 6890N) coupled to a quadripolar mass spectrometer (Agilent 5973N) was used with ionization by electronic impact (70 eV). The apparatus was fitted with a HP-5MS column (internal diameter: 0.25 mm, length: 30 m, film thickness: 0.25 *μ*m). The carrier gas was helium at a flow of 0.5 mL/min. The injector port temperature was 250°C and split ratio was 20 : 1. The transfer line temperature was 280°C, ion-source-heating was 230°C, and the scan-range was 40–700* m/z*. The GC oven program was as follows: 40°C held for 5 min and then ramped at 4°C/min to 300°C, with a final hold time of 10 min. Interpretation and identification of the fragmentation mass spectrum was carried out by comparison with the Wiley NBS mass spectrum data base. Results were expressed as the relative percentage of peak area in the chromatogram.

### 2.8. Preparation of Liposomes (LipoRHIC and LipoRHIWC)

Liposomes were prepared as previously described by Sinico et al. [21] with some modifications. Briefly, 5.0 mg of hexane fractions (hexRHIC or hexRHIWC), 20 mg of phosphatidylcholine (Sigma Aldrich), 2.6 mg of cholesterol (Sigma Aldrich), and 0.3 mg of Tween 20 (Sigma Aldrich) were dissolved in 10 mL of chloroform (Tedia) in a 50 mL round-bottomed flask. The solvent was removed by evaporation, and a lipid film was formed on the inner wall of the flask. The lipid film obtained was hydrated with 2.0 mL of distilled water and a combination of both sonication and homogenization was performed for 30 min (5.0 min intervals) at room temperature for better drug loading. Finally, liposomes of the turmeric cortex (LipoRHIC) and of the turmeric without cortex (LipoRHIWC) were separated by ultracentrifugation at room temperature and 15,000 rpm for 7.0 min and filtered through a 0.20 *μ*m filter (Whatman). LipoRHIC and LipoRHIWC were stored at 4°C for no longer than 15 days.

### 2.9. Determination of Entrapment Efficiency of LipoRHIC and LipoRHIWC

The entrapment efficiency of the liposomes hexRHIC and hexRHIWC was calculated in relation to turmerone (Sigma Aldrich) present in the vesicles. This percentage encapsulated was determined after lysis of the prepared liposomes with ethanol/chloroform (1 : 1) and sonication for 10 minutes. The concentration of turmerone in the liposomes was determined spectrophotometrically at 235 nm using a UV-visible spectrophotometer (model UV-601 PC, Shimadzu). This procedure was performed three times. A calibration curve was traced with five levels of triplicate analysis of standard solutions containing turmerone at concentrations of 0.5 to 3.0 mg. Samples and standard solutions were diluted in methanol. The equation of the regression (Absorbance = 0.061 × Concentration) showed linear fit in the concentration range studied (*R* ^2^ = 0.996). Variations among analyzes of replicates were less than 0.1%. Blanks containing liposomes without turmerone were evaluated under the same conditions and did not show any absorbance at 235 nm. The entrapment efficiency was expressed as follows: entrapment percentage (EP%) = entrapment sample related to turmerone/total turmerone × 100.

## 3. Results and Discussion

Curcuminoids isolated from* Curcuma longa *have a long history as antimicrobial agents, including against* Leishmania* [22, 23]. Although there are various reports about curcuminoids as the main molecular targets of* Curcuma* species, other constituents can be considered unique or coadjuvant molecular target of the curcuminoids in different therapies [24]. The essential oil of turmeric has been pharmacologically studied as an anti-inflammatory, anticancer, and antimicrobial agent among other activities. Its main constituents, associated to various species of* Curcuma*, are sesquiterpenoids like turmerones [25–30]. In this work, the hexane fractions (hexRHIC and hexRHIWC) obtained from the crude methanol extract of the turmeric cortex and turmeric without cortex of* C. longa*, respectively, were analyzed by GC-MS and the constituents are listed in Table 1. All substances detected in the extracts have already been described for the* Curcuma* species [31–34]. The comparison of the chromatographic profiles of the fractions, hexRHIC and hexRHIWC, showed a similarity in the composition, and the main difference was the relative percentage of each constituent. The main constituents of both fractions were the turmerones with total amounts of 40.50 and 30.80%, respectively.

The antileishmanial activity of the hexRHIC and hexRHIWC extracts from* C. longa* was evaluated against the promastigote forms of* L. amazonensis*. These fractions showed activity against the strain at minimal inhibitory concentrations of 125 and 250 *μ*g/mL (IC50 = 35.4 and 83 *μ*g/mL), respectively. Considering that ar-turmerone is one of the major components of both fractions, it was deemed necessary to test the antileishmanial activity of this substance alone. The MIC value of ar-turmerone was 50 *μ*g/mL (IC_50_ = 11 *μ*g/mL) against the promastigote forms of* L. amazonensis*. This result showed that it was more active than the hexane extracts and could be considered one of the target substances against* Leishmania*.

**Figure 1:**
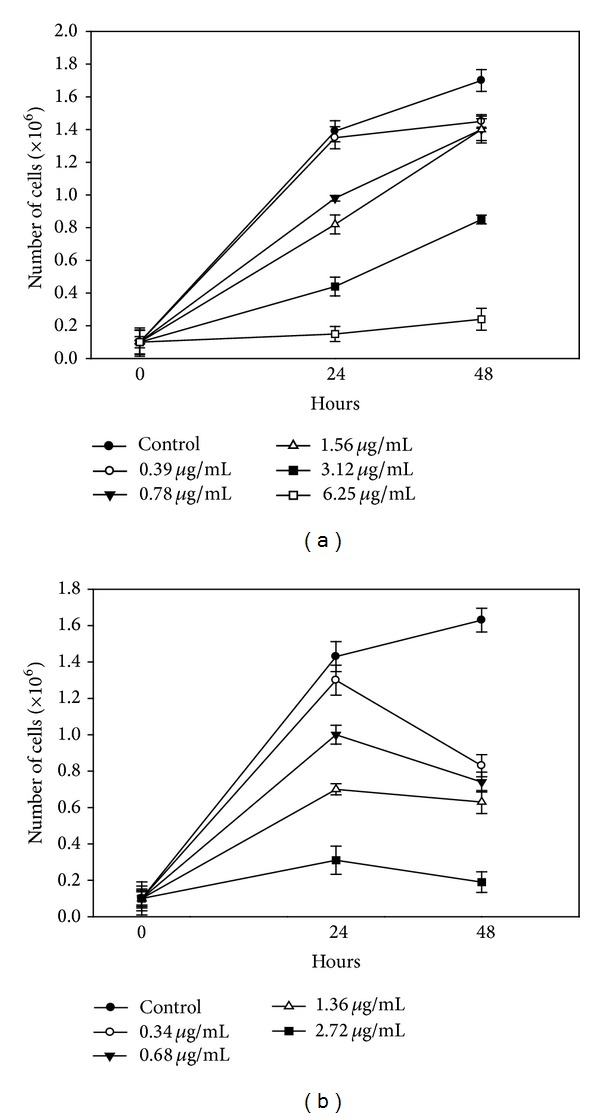
Growth inhibition effect of hexane fractions in liposomal formulations on* L. amazonensis* promastigotes. Parasites were counted at 24-hour intervals using a Neubauer chamber. (a) Promastigotes treated with LipoRHIWC; (b) promastigotes treated with LipoRHIC. Each bar represents the mean ± standard error of at least three independent experiments, which were performed in duplicate.

Solubility can be a problem when plant extracts and fractions are assayed, especially those with nonpolar characteristics, considering the need to disperse them in an aqueous media. Low solubility can influence the bioactivity of these samples directly by decreasing it or even inhibiting it completely. During the assays, some material precipitation was observed in the samples, which could be associated with the low activity of the extracts. Recently, much attention has been given to the search of novel drug delivery systems for drug-candidates in the combat of leishmaniasis. Liposomes as a drug delivery system have an interesting approach, because they are able to reduce the toxicity, prolong the action, and improve biodistribution and the stability of the drugs [35]. Thus, in order to improve solubility and antileishmanial activity of the* C. longa* extracts, liposomes, LipoRHIC, and LipoRHIWC, were prepared from the hexRHIC and hexRHIWC extracts and tested against* L. amazonensis* promastigotes. The percentage entrapment efficiency of the liposomes based on turmerones was 46.5% and 43.6%, respectively, and an increase in the activity of the two fractions was observed. However, attempts to incorporate turmerone in the liposomal form are not promising because of the volatility of this sesquiterpenoid. Subinhibitory concentrations (sub-MIC) of LipoRHIC and LipoRHIWC were able to affect parasite growth after 48 h treatment.  Figure 1(a) shows a strong inhibition of the promastigote forms in the presence of LipoRHIWC at 6.25 *μ*g/mL (IC_50_/48 h = 2.9 *μ*g/mL), when compared to control parasites. Despite the antileishmanial activity of LipoRHIWC, better results were observed when parasites were treated with LipoRHIC (Figure 1(b)). After 48 h treatment, LipoRHIC inhibited parasites growth at 2.75 *μ*g/mL (IC_50_/48 h = 0.4 *μ*g/mL). The combination of the plant fractions and liposomal formulations has been successfully described in other studies. Lupane, a triterpene isolated from* Combretum leprosum* previously described as a leishmanicidal agent against* L. amazonensis* promastigotes [36], was recently incorporated into liposomes in a study conducted by Barros et al. [37]. Liposomal-lupane (6.0 *μ*g/mL) reduced the number of parasites in murine macrophages by 61.7%. Another strategy using liposomes against a protozoan infection was demonstrated by Aditya et al. [38] with curcuminoids obtained from* C. longa* loaded into soybean phosphatidylcholine. These liposomes were able to reduce parasitemia and increase the survival of murine models with a* Plasmodium berghei* infection. Lala et al. [39] reported the antileishmanial activity of several vesicular delivery systems, including liposomes, incorporated with *β*-carboline alkaloid (harmine) isolated from* Peganum harmala*. Free harmine displayed an effective (IC_50_) dose of about 25 *μ*g/mL against* L. donovani* promastigotes, but when incorporated into liposomes and administered to infected hamsters, it showed a decrease of about 61% of the parasite load in the spleen when compared with untreated animals.

**Figure 2:**
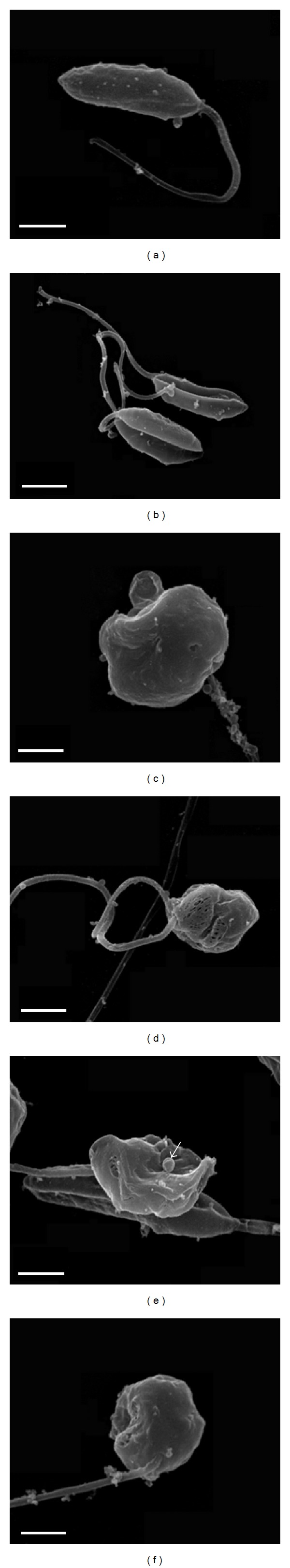
Morphological alterations of* L. amazonensis* promastigotes treated with LipoRHIC. ((a)-(b)) Untreated parasites; (a)* L. amazonensis* displaying the characteristic morphology of promastigotes; (b) promastigotes under mitotic process; ((c)-(d)) promastigotes treated with LipoRHIC liposomal at MIC concentration (5.5 *μ*g/mL) for 24 h; (c) parasite showing “blebs” scattered over the flagella and rounded shape; (d) promastigotes under abnormal mitotic process, significant cell shrinkage and flagella reduced in size. Note the presence of pores on membrane surface; ((e)-(f)) promastigotes treated with LipoRHIC at a sub-MIC concentration (2.75 *μ*g/mL); (e) some parasites showing “blebs” scattered over the plasma membrane (arrow); (f) parasite showing rounded shape. Bars = 1 *μ*m.

**Figure 3:**
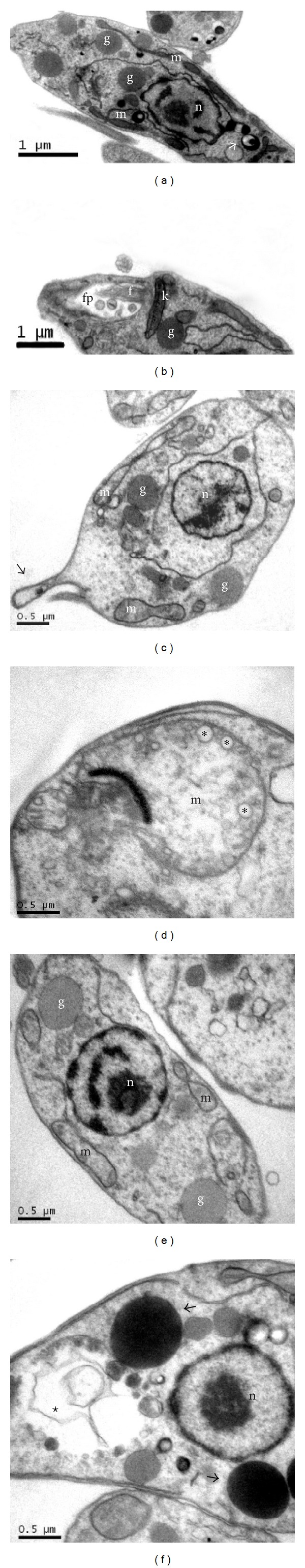
Ultrastructure alterations induced by LipoRHIC in* L. amazonensis *promastigotes. ((a)-(b)) Sections of untreated promastigote forms showing the main structures observed under transmission electron microscopy. (a) Promastigote presenting typical elongated body. The nucleus is rounded and the mitochondrion is branched. White arrow shows acidocalcisome in the cytoplasm; (b) normal flagellum and flagellar pocket are observed in the anterior portion of the parasite. Mitochondrion containing the kinetoplast can also be noted. ((c)–(f)) Parasites treated for 24 h with MIC concentration (5.5 *μ*g/mL) of LipoRHIC. (c) Parasite presenting rounded body and an abnormal membrane projection (black arrow); (d) detail of mitochondrion swelling and the presence of several vacuoles (*); ((e)-(f)) complete intracellular disorganization although the nucleus membrane remained intact. (f) Autophagic structure (★) and electron-dense granules (black arrows). n: nucleus; m: mitochondrion; k: kinetoplast; f: flagellum; fp: flagellar pocket; g: lipid.

Since LipoRHIC was the most effective against* L*.* amazonensis* promastigotes, cellular alterations of these parasites were evaluated through scanning and transmission microscopy. Parasite photomicrographs revealed serious morphological alterations after 24 h exposure to LipoRHIC (Figure 2). At the MIC concentration (5.5 *μ*g/mL), promastigotes showed “blebs” scattered over the flagella and rounded shapes could be observed (Figure 2(c)) in contrast to untreated parasites (Figure 2(a)-2(b)). LipoRHIC-treated promastigotes displayed significant alterations during the mitotic process, including cell shrinkage and flagella reduced in size (Figure 2(d)). Furthermore, even when parasites were treated with a subinhibitory concentration (2.75 *μ*g/mL) for 24 h, the alterations, such as membrane “blebs” and rounded bodies, became visible (Figure 2(e)-2(f)). In addition, the effects of LipoRHIC against* L*.* amazonensis* promastigotes were observed using transmission electron microscopy. Ultrastructural changes in promastigotes treated with LipoRHIC at the MIC concentration (5.5 *μ*g/mL) showed significant alterations (Figure 3). After 24 h of treatment, parasites displayed a rounded body and had an abnormal membrane projection (Figure 3(c)) and mitochondrion swelling with the presence of several vacuoles inside the organelle (Figure 3(d)). Figures 3(e) and 3(f) show that most of the cells presented complete intracellular disorganization, as well as autophagic structures at the end of the treatment. Similar mitochondrion alterations and the presence of autophagic structures have been reported by previous studies as a possible consequence of sterol biosynthesis inhibition [40–43]. Some enzymes belonging to the Trypanosomatidae family that directly participate in sterol biosynthesis, such as Δ^24(25)^-sterol methyltransferase, are not expressed in mammalian cells [44]. Therefore such enzymes could be interesting targets for new drug candidates against trypanosomatids, including* Leishmania* [45].

In conclusion, despite the fact that the antileishmanial activity of curcuminoids has been extensively studied by various laboratories worldwide, the results reported here highlight the positive influence of the volatile constituents (enriched in turmerones) not hitherto associated with antileishmanial activity, reinforcing the scientific evidence of* C. longa* as the botanic species of the century. In addition, the incorporation of the hexane fractions into liposomes was demonstrated to be an interesting approach for the study of new antileishmanial agents from natural sources.

## Figures and Tables

**Table 1:** Chemical composition of *C. longa *hexane fractions.

Compounds	RI^lit.^	RI^cal.^	HF composition (%)	
			Turmeric cortex	Turmeric without cortex
*trans*-*β*-Farnesene	1456	1450	—	0.4
ar-Curcumene	1480	1476	4.5	5.0
*α*-Zingiberene	1493	1489	13.6	24.1
*β*-Bisabolene	1505	1504	2.0	3.3
*β*-Sesquiphellandrene	1522	1519	12.5	16.3
*E*-*iso*-*γ*-Bisabolene	1529	1525	0.9	0.8
ar-Tumerone	1669	1667	15.8	14.6
*β*-Turmerone	1677	1678	17.5	10.7
Germacrone	1693	1690	6.8	4.9
Curlone	1701	1699	7.2	5.5
Curcumenol	1734	1731	0.7	0.6
(6R,7R)-Bisabolone	1742	1741	2.1	1.9
Dehydrocurdione	1891	1889	6.8	5.3
Identified components			90.4	93.4

RI^cal.^: retention index based on a homologous series of normal alkanes. RI^lit.^: retention index from literature. HF: hexane fractions.
